# Improving antimicrobial use through antimicrobial stewardship in a lower-middle income setting: a mixed-methods study in a network of acute-care hospitals in Viet Nam

**DOI:** 10.1016/j.jgar.2021.09.006

**Published:** 2021-12

**Authors:** Vu Thi Lan Huong, Ta Thi Dieu Ngan, Huynh Phuong Thao, Nguyen Thi Cam Tu, Truong Anh Quan, Behzad Nadjm, Thomas Kesteman, Nguyen Van Kinh, H Rogier van Doorn

**Affiliations:** a Oxford University Clinical Research Unit, 78 Giai Phong, Hanoi, Viet Nam; b National Hospital for Tropical Diseases, 78 Giai Phong, Hanoi, Viet Nam; c Hospital for Tropical Diseases, 764 Vo Van Kiet, Ho Chi Minh City, Viet Nam; d MRC Unit The Gambia at The London School of Hygiene & Tropical Medicine, Fajara, The Gambia; e Centre for Tropical Medicine and Global Health, Nuffield Department of Medicine, University of Oxford, Oxford, UK

**Keywords:** Antimicrobial stewardship, Antimicrobial prescribing, Antimicrobial resistance, Low- and middle-income countries, Viet Nam

## Abstract

**Objectives:**

This study aimed to analyse the current state of antimicrobial stewardship (AMS) in hospitals in Viet Nam, a lower-middle income country (LMIC), to identify factors determining success in AMS implementation and associated challenges to inform planning and design of future programmes.

**Methods:**

We conducted a mixed-methods study in seven acute-care hospitals in the antimicrobial resistance (AMR) surveillance network in Viet Nam. Data collection included 7 focus group discussions, 40 in-depth interviews and a self-administered quantitative survey of staff on AMR and AMS programmes. We summarised qualitative data by reporting the most common themes according to the core AMS elements, and analysed quantitative data using proportions and a linear mixed-effects model.

**Results:**

The findings reveal a complex picture of factors and actors involved in AMS implementation from the national level to the departmental and individual level within each hospital. The level of implementation varied, starting from the formation of an AMS committee, with or without active delivery of specific interventions. Development of treatment guidelines, pre-authorisation of antimicrobial drug classes, and post-prescription audit and feedback to doctors in selected clinical departments were the main interventions reported. A higher level of leadership support and commitment to AMS led to a higher level of engagement with AMS activities from the AMS team and effective collaboration between departments involved.

**Conclusion:**

Establishing country-specific guidelines on AMS staffing and adapting standards for AMS education and training from international resources are needed to support capacity building to implement AMS programmes effectively in LMICs such as Viet Nam.

## Highlights

• Antimicrobial stewardship (AMS) in low- and middle-income settings has multiple players.
• Leadership commitment greatly influences AMS implementation in hospitals.
• Staff showed good perception of AMS but misperceptions on local resistance levels.
• Guidelines on staffing and training standards are needed to support implementation.
• More support is needed to promote active roles of pharmacists and microbiologists.

## 1 Introduction

Antimicrobial stewardship (AMS) is an important approach to improving antimicrobial use and controlling antimicrobial resistance (AMR) in clinical settings. Several guidelines and recommendations on AMS implementation in hospitals are available [1], [2], [3], [4], [5] and a number of reviews have demonstrated the effectiveness and potential economic impact of these programmes worldwide [6], [7], [8], [9], [10] and in Asia [11,12]. Notable differences exist between and among Asian and other high-income and low- and middle-income countries (LMICs) that can mediate how AMS programmes are implemented in the Asian context, including healthcare system and insurance, doctor–patient communication, perceptions about antibiotics, and hygiene practices [11,13].

Improving antimicrobial use through AMS is among the priority strategies of the AMR National Action Plan 2013 in Viet Nam, and this was first introduced to a network of 16 hospitals within the Viet Nam Resistance Project (VINARES) in the same year [14]. A national AMS guideline was issued in 2016 [15] and was updated in 2020 [16] to provide a framework for hospitals to implement AMS activities. The guideline recommends each hospital to establish a multidisciplinary AMS committee led by the hospital director board with members from relevant departments including planning/administrative, clinical (e.g. internal medicine, surgery, critical care, infectious diseases), pharmacy, microbiology, quality assurance and control, infection prevention and control (IPC), and information technology.

A recent review by the Ministry of Health (MoH) Viet Nam in 2018 showed that 334 (51%) of 655 hospitals had established an AMS team consisting of a pharmacist (90%), staff of the planning department (79%), internal medicine doctor (76%), nurse (67%), surgical doctor (65%), IPC staff (64%) and microbiologist (39%) [17]. Microbiology was available in 332/655 (51%) hospitals, 47% of which performed antimicrobial susceptibility testing and 33% of which had hospital-wide antibiograms. Specific interventions among the 655 hospitals included facility-specific treatment guidelines (22%), pre-authorisation policy (41%), clinical microbiology guidelines (29%) and basic infection control policy (70%). Most hospitals had not developed assessment criteria for AMS indicators on antibiotic use (58%), hospital-acquired infections (6%), guideline compliance (67%) and AMR levels (83%).

AMS implementation requires cultural and behavioural changes and is complex [18], [19], [20]. Studies have identified factors facilitating or hindering implementation, but limited information is available from LMIC settings. A review summarising the key challenges included lack of access to diagnostics, quality drugs and supporting infrastructures as well as limited knowledge of healthcare staff [21]. The World Health Organization (WHO) developed a toolkit for AMS programmes in LMICs that can be used to guide implementation depending on the resources available [5]. Nevertheless, understanding the determinants of success or failure is crucial for further steps to improve the uptake and continuation of AMS programmes across hospitals in a LMIC such as Viet Nam. This study analysed factors determining success in AMS implementation as well as any associated challenges based on a mixed-methods approach at hospitals within the VINARES network.

## 2 Methods and materials

### 2.1 Study setting

This mixed-methods study was conducted in 2018 at seven hospitals in the VINARES network. The VINARES network originally consisted of 16 hospitals equipped with microbiology laboratories, including 4 national, 7 provincial and 5 specialised hospitals (infectious diseases, surgical, paediatric). The network was set up in 2012 to conduct surveillance of antibiotic use and resistance and to provide impetus and tools for developing AMS programmes in hospitals in response to the situation analysis on antibiotic use and resistance in Viet Nam [14].

### 2.2 Study design and data collection

This study involved multiple data collection methods, including in-depth interviews (IDIs), focus group discussions (FGDs) and a quantitative survey to assess participants’ experience with AMS implementation and prescribing practices. We preselected ten hospitals within the VINARES network (three national, four specialised and three provincial hospitals) and geographical regions (five in the north, two in the centre and three in the south of the country).

Data collection was piloted at one hospital (two IDIs, one FGD and survey form). Main data collection was conducted at two hospital groups: Group 1 with five hospitals (one national, two specialised and two provincial) where no specific AMS actions had been identified; and Group 2 with four hospitals (two national, one specialised and one provincial) where an AMS programme had been initiated. Group classifications were based on the information gathered from our previous experience and communication with the local hospitals and partners.

In each hospital of Group 1, we conducted two FGDs (one with senior and one with junior doctors); FGD was chosen to provide a group setting to stimulate discussion among staff and to generate more information as each individual staff might have limited experience about AMS implementation. In each hospital in Group 2, we planned to conduct 12 IDIs with hospital staff involved in AMS implementation in relevant departments to collect more in-depth individual experience in a non-threatening environment.

We developed semi-structured IDI and FGD question guides based on the key components described in the national AMS guidelines, which were also in accordance with the US Centers for Disease Control and Prevention (CDC) checklist [22] and the global core elements [23]. After piloting, only minor revisions were made to the original question guides (Supplementary methods). The IDIs and FGDs focused on: antimicrobial prescribing practices; involvement and collaboration between clinical wards, microbiology and pharmacy department in antimicrobial treatment and AMS programme implementation; and factors influencing AMS implementation. Participants of IDIs and FGDs were also asked to complete a quantitative survey about their perceptions and attitudes towards AMR, antimicrobial prescribing practices and the implementation of AMS activities at the hospital (see Supplementary methods).

### 2.3 Data analysis

Audio recordings of IDIs and FGDs were transcribed and uploaded to NVivo v.12 [24] for data management and analysis. Data were analysed through systematic coding and indexing and themes were identified for data interpretation. We identified common themes discussed throughout the IDIs and FGDs and analysed the qualitative data by hospital groups (Table 1). We summarised data by reporting the themes that were most frequently mentioned by the participants, and describing the implementation status according to the core AMS elements: leadership; accountability and responsibilities; expertise on infection management; education and training; other specific actions aimed at responsible antimicrobial use; monitoring and surveillance; and reporting and feedback [23]. We identified the actors influencing doctors’ prescribing practices and implementation of AMS programmes in the study hospitals based on the framework reported in a review on AMS strategy by Dyar et al. [25]. Data from the quantitative survey on perceptions and attitudes were summarised and visualised using a Likert scale [26] for each survey statement by each hospital group to describe similarities and differences between the groups.

**Table 1:** Summary of hospitals and qualitative data collection methods participating in the study

Hospital type	Bed capacity	Group	Method	Participating departments									
				ED	ICD	ICU	IDD	IMD	MIL	OPD	PHD	PED	SUD
National, specialised	<1000	2	2 IDIs, 1 FGD	x	x	x		x	x		x		
Provincial, specialised	<1000	2	13 IDIs		x	x		x	x	x	x	x	
National, general	>2000	2	13 IDIs			x	x	x	x		x	x	
Provincial, general	2000	1	12 IDIs			x	x	x	x		x		x
National, general	1000	1	2 FGDs	x		x		x	x		x		x
Provincial, general	>2000	1	2 FGDs		x	x		x	x		x		
Provincial, general	1000	1	2 FGDs	x		x		x	x		x	x	x

ED, emergency department; FGD, focus group discussion; ICD, infection control department; ICU, intensive care unit; IDD, infectious diseases department; IDI, in-depth interview; IMD, internal medicine department; MIL, microbiology laboratory; OPD, outpatient department; PED, paediatric department; PHD, pharmacy department; SUD, surgical department.

To assess the level of awareness of healthcare staff about AMR in the local settings, they were asked to evaluate the proportion of resistant organisms for nine ‘bug–drug’ combinations. We then compared these proportions with those reported in the AMR surveillance system of each hospital and computed, for each participant and bug–drug combination, the difference between perceived and measured proportions, as expressed in percentage points. We used a linear mixed-effects model (with *lmer* function in *lmerTest* package in R program v.4.0.0) to examine the factors associated with this difference, with individual participants and hospitals included as a random effect.

## 3 Results

We contacted and invited ten hospitals, of which seven agreed to participate in this study. These included two national (Hue Central General Hospital, Hue; Can Tho Central General Hospital, Can Tho), three provincial (Uong Bi Viet Nam-Sweden Hospital, Quang Ninh; Viet Tiep Hospital, Hai Phong; Da Nang Hospital, Da Nang) and two specialised (National Hospital for Tropical Diseases, Ha Noi; Hospital for Tropical Diseases, Ho Chi Minh). The three hospitals that did not participate included one national hospital that previously reported to have an active AMS programme and two specialised hospitals (surgical and paediatric) with no information on AMS implementation at their institution. As these were national hospitals with unique characteristics, we could not find any other hospitals in the network for replacement.

The process of conducting IDIs and FGDs followed the same study procedures in the pilot phase as the main data collection phase and since there was no major revision to the pilot IDI and FGD question guide and format, data collected during this pilot phase was also included in the data analysis. Based on the data collected from IDIs and FGDs, we verified the accuracy of the classification of the hospitals into two groups: Group 1 consisting of four hospitals without an active AMS programme; and Group 2 consisting of three hospitals with an active AMS programme implemented by the time of data collection. One provincial hospital was considered to have an active AMS programme based on our prior information, however data from the IDIs showed a lack of active implementation and therefore was grouped to Group 1 in the analysis (Table 1).

### 3.1 Factors and actors involved in hospital antimicrobial stewardship implementation

**Fig. 1:**
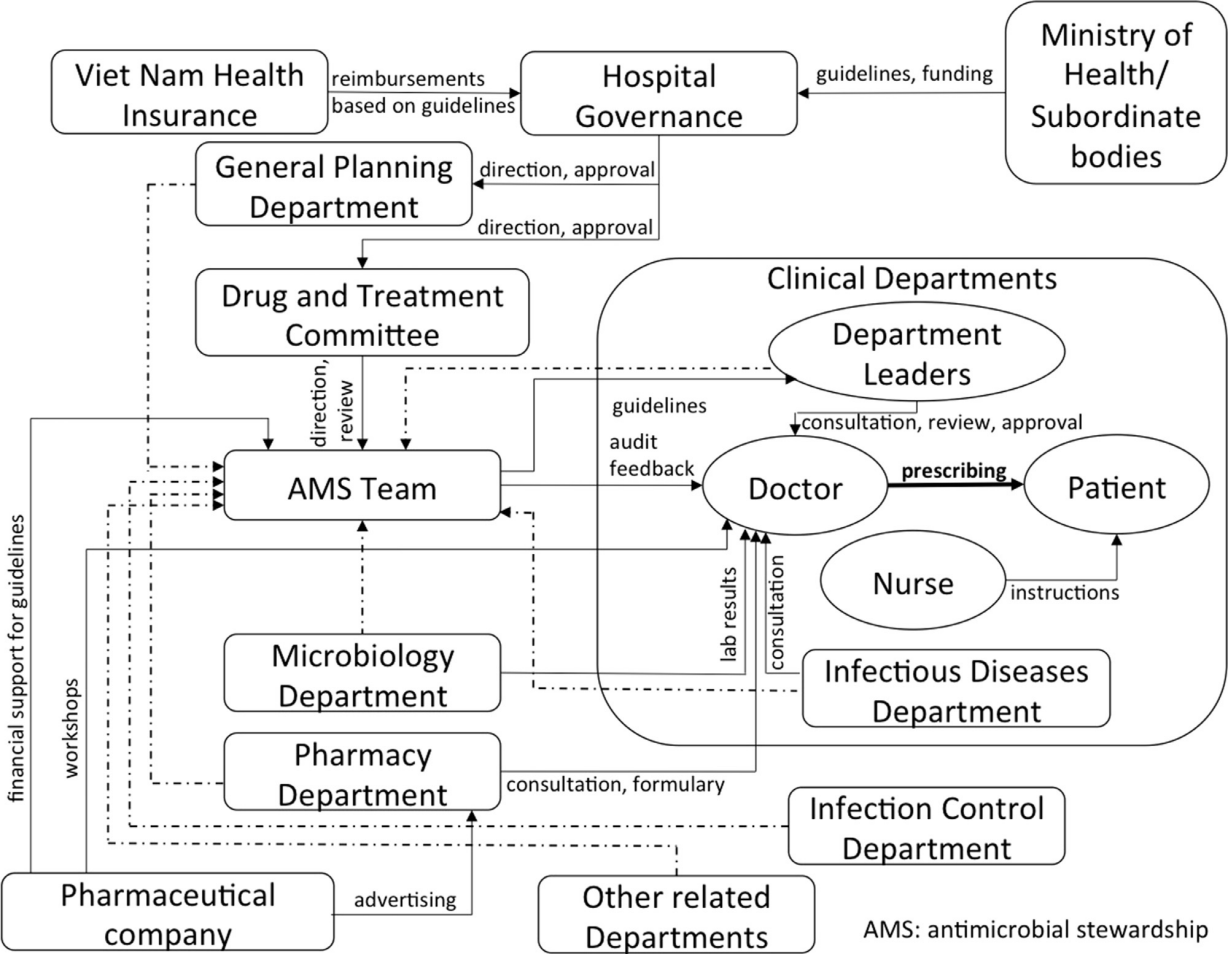
Summary of main actors influencing doctors’ prescribing practices for antimicrobial drugs and implementation of antimicrobial stewardship (AMS) programmes in the study hospitals. Solid arrows indicate direct influence, and dotted arrows indicate participating in the AMS team.

**Fig. 2:**
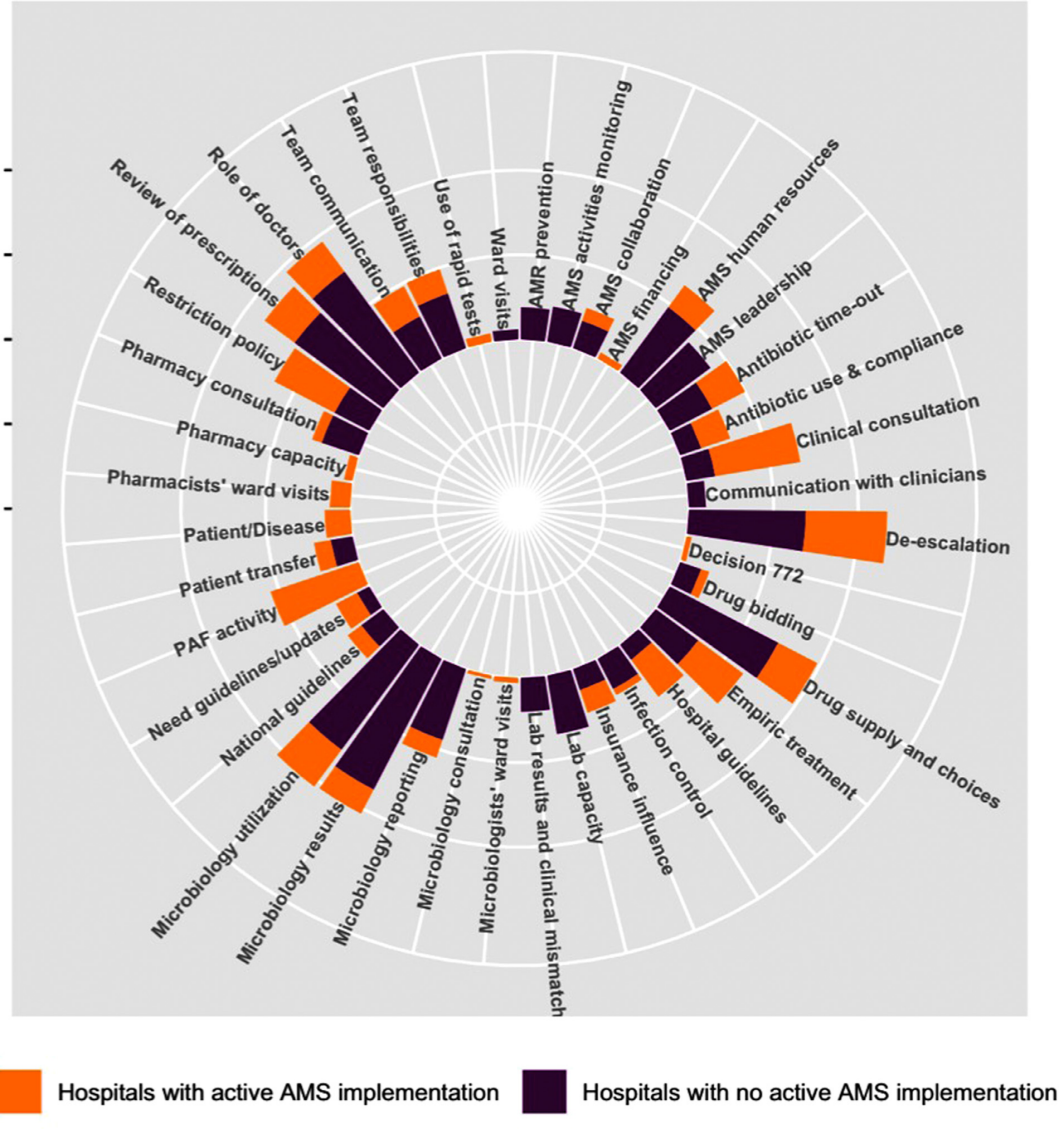
Frequency of the main themes regarding antibiotic prescribing and antimicrobial stewardship (AMS) implementation discussed by the participants in two hospital groups. Bars represent the number of times each theme emerged in the transcripts, with each scale interval corresponding to a frequency of 50. AMR, antimicrobial resistance; PAF, prospective audit and feedback to prescribers.

A hospital AMS programme involves a number of actors and processes that can influence doctors’ practices of prescribing antimicrobial drugs (Fig. 1). These included actors from the national level (MoH, health insurance), hospital level (governing units, departments), as well as external stakeholders (pharmaceutical companies) and individuals (doctors, nurses, patients). Fig. 2 describes the factors associated with prescribing practices and AMS; similar themes were found between Groups 1 and 2. Common aspects discussed were microbiology service-related factors (low utilisation, suboptimal quality of results and long turnaround time), role of doctors (clinical experience, seniority and position) and drug supply and choices (sources) for participants in Group 1, and consultations (with clinical specialty, microbiology and pharmacy) and AMS interventions (restriction policy and review of prescriptions) in Group 2. In both groups, lack of confidence in and long turnaround time for microbiology results as well as drug quality were thought to reduce doctors’ confidence in de-escalating antibiotic treatment. The overall attitude was not to de-escalate if a patient still responded to the current treatment or until they were transferred to another department (see representative quotes in Table 2 and additional quotes in Supplementary Table S1):

*“Due to high risk of hospital acquired infections and cross-infection, we are hesitant to de-escalate… Most drugs are generic, we do not feel sure about the drug quality so not confident to de-escalate.” (FGD, junior staff)*

**Table 2:** Summary of implementation status and representative quotes about key activities as part of the antimicrobial stewardship (AMS) programme in the studied hospitals

Activity	Summary	Representative quotes
Guidelines	Two specialised hospitals (Group 2) developed and regularly updated antimicrobial treatment guidelines based on local microbiological evidence and integrating risk stratifications for infection with multidrug-resistant organisms and hospital-acquired infections. These guidelines were available also in a mobile app and handbook, and in one hospital integrated into the hospital information system. In other hospitals, doctors used the national guideline and guidelines from nationally recognised hospitals. The national guideline was criticised for a lack of details for specific clinical conditions	“*The guideline by the Ministry of Health and our actual clinical practices are too different, I have to say frankly. It is now more for insurance purposes and needs to be more practical.*” (FGD, senior staff)
Education activity	At one hospital with active implementation, at the beginning of the programme, some key AMS staff were sent for international training on stewardship in Taiwan. At this hospital, training on hospital-specific antimicrobial treatment guidelines was provided to staff regularly to increase awareness and compliance. For other hospitals, staff only attended ad hoc workshops and training on related topics organised by external partners including pharmaceutical companies	“*Training on antimicrobial drugs and AMR should be more regular for us to update our knowledge. I remember I had some training two or three years ago, so not so often.*” (IDI, respiratory internist, female, 33 years old)
Building IT and data capacity for AMS activities	There was a lack of IT capacity to support AMS activities in all hospitals, except for the hospital with the most active programme. Routine data were not summarised to provide regular feedback to doctors on AMR and antibiotic use. AMR data summary was reported to the hospital level on a semi-annual or annual basis. Hospitals also submitted annual reports on antimicrobial consumption and antimicrobial susceptibility to the MoH	“*I think the use of electronic medical records should be implemented because patient stratification prior to treatment is very important. Secondly, it would make things easier for us as pharmacists in controlling or approving drugs for patients because it would contain more information. Now we are still using paper medical records, we can't visit each department to approve drugs for patients, so it is very difficult*.” (FGD, junior staff at a 1000-bed general hospital with four clinical pharmacists)
Pre-authorisation	This process was accomplished by the treating doctor calling for a higher-level consultation (department level or hospital level) and seeking approval prior to use for treatment with restricted antibiotics by head of the clinical department, clinical pharmacist (if available), head of pharmacy and director board representative. However, there were shortcuts in the procedure and restricted drugs were still used before consultations and approval	“*Yes, we have tried to follow the procedure, and we reminded doctors frequently. However, sometimes this was done only to meet the requirements, the treating doctor checked with the chief doctor during the night shift to have the approval later. In principle, we need to have consultations before approval and use but in reality this could not be done all the times, so they prescribed and left the form blank for signature later.*” (IDI, quality control/emergency doctor, male, 35 years old)
Audit and feedback	Review of antimicrobial prescriptions was done prospectively or retrospectively at three hospitals. Review results were summarised in reports and communicated back to head of reviewed departments in hospital-wide or departmental meetings. Prospective review was conducted most rigorously at an infectious diseases hospital, and at this hospital advice from the AMS team for the prescribers was easily accessible to the prescribing doctors. Prospective review was also partially adopted at one national general hospital at a few clinical wards but was limited due to lack of staff capacity	“*Currently we have six clinical pharmacists (>2000-bed hospital), but there is too much work including drug procurement and authorisation, so we have not got much time for clinical visits. We only focus on some wards with high antimicrobial use including ICU… This is done on a weekly basis, mainly to check if drugs have been approved before use and if they were used appropriately.*” (IDI, clinical pharmacist, female, 54 years old)
Documentation of treatment	All treatment plans including antibiotic treatment for each patient were to be specified on a daily basis following the MoH documentation format. Doctors usually document the drugs and reasons for using the selected treatment, but not the planned date for review or stop. The frequency of reviewing treatment plans by doctors depended on clinical severity: around 24 h for severe patients and 2–3 days for non-severe patients	“*We usually write in the medical records that culture results were returned with the AST results and that we adjusted antibiotics based on the AST results… We treat with the duration based on what we learned, we don't write the anticipated stop date.*”(IDI, ICU doctor, male, 32 years old)
Monitoring and reporting	Microbiology and pharmacy department made summary reports annually or every 6 months to the management board and for the MoH. These were usually fed-back to heads of departments through emails but not directly to individual prescribers. Appropriateness of prescriptions was not monitored regularly in five hospitals. One significant difficulty was a lack of IT capacity to support AMS activities, and pharmacy staff have been struggling with monitoring patient antimicrobial prescriptions with the current paper-based system	“*In this AMS program, the responsibilities of clinical pharmacists are very high. When reading the guideline, I felt very tired, surveys, evaluations, guidelines and other things… with only the pharmacy in charge. The pharmacy department does not have many staff. The clinical pharmacists now just focus on the bidding process, and rarely visit the wards for clinical work.*” (FGD, senior staff) “*No evaluation here because doctors are hesitant about appropriateness of prescriptions being monitored and there are insufficient clinical pharmacists available to monitor*.” (FGD, senior staff) (Both quotes from the same FDG at >2000-bed hospital with six clinical pharmacists)

AMR, antimicrobial resistance; AST, antimicrobial susceptibility testing; FGD, focus group discussion; ICU, intensive care unit; IDI, in-depth interview; IT, information technology; MoH, Ministry of Health Viet Nam.

Group 1 more frequently discussed issues related to AMS implementation, including the need for human resources, strong leadership support, and clear definitions of responsibilities for AMS staff. Group 2 more frequently discussed guidelines and pharmacy-related aspects because there were more activities in guideline development and use and pharmacy involvement in AMS in these hospitals. Pharmacists in Group 2 implemented reviews of antimicrobial prescriptions retrospectively or prospectively (frequency varied from weekly to once every few months), while in Group 1 pharmacists only joined clinical consultations on request from doctors and were more involved in drug procurement procedures than patient care.

### 3.2 Assessment based on core elements

#### 3.2.1 Leadership

The national AMS guideline was considered important guidance for implementing AMS in hospitals. Leadership commitment and support was an essential condition for an effective AMS programme:

“*Lack of resources is not unique to any hospital. It is more important that there is leadership consensus and support and united commitment of the staff for the stewardship programme… We have enough guidance from the MoH, and we can learn from the models from other hospitals.” (IDI, microbiology, female, 36 years old)*

#### 3.2.2 Accountability and responsibility

Five hospitals established a separate AMS committee, one formed an AMS subcommittee under the existing Drug and Treatment Committee, and one did not establish an AMS committee in their hospital. Participants in the hospital without an AMS committee expressed the need for establishing this to help improve their antimicrobial prescribing practices. The composition of AMS committees/subcommittees followed national guidelines, which were led by a representative of the hospital director board and included members from all relevant departments. One common concern raised in the hospitals with an AMS committee/subcommittee was that no full-time staff was responsible for AMS, even for the core staff co-ordinating the programme:

*“Our core AMS team includes ten staff with multiple responsibilities including their core job responsibilities. Most of these are part of the hospital director board. How can they have time to check medical records? So we need to have treating doctors to help review medical records.” (IDI, AMS co-ordinator/pharmacy, female, 34 years old)*

#### 3.2.3 Expertise on infection management

Each hospital not specialised in infectious diseases had a department of infectious diseases/tropical medicine. These doctors are often called in when there are complicated cases with signs of infection. There was good collaboration between clinical departments, and all participants commented positively on the access to infectious diseases consultations.

Quality-controlled microbiology services are available at all study hospitals with support from several international organisations including Oxford University Clinical Research Unit (OUCRU) and the CDC. However, the level of use and quality of services varies greatly, with higher data use and engagement in AMS at two specialised hospitals for infectious diseases. At one of these two hospitals, the chief clinical microbiologist was actively involved in providing consultations for patient antimicrobial treatment (usually through daily phone calls). Among other hospitals, lack of trust in sample collection (which is usually performed by nurses at the ward) and therefore culture results as well as lack of communication between microbiology and clinical doctors contributed to the low use of microbiology services:

*“Interaction between microbiology and clinical doctors is currently very weak. In many cases, microbiology results do not correspond to the clinical symptoms of patients, possibly due to contamination in specimen collection.” (IDI, quality control/emergency, male, 35 years old)*

*“We can tailor our treatment based on susceptibility results, however, we need to consider the fact that not all specimens are taken correctly and therefore ask whether we can trust the results of susceptibility testing.” (FGD, junior staff)*

Interactions between pharmacists and clinical doctors occurred mostly as consultations for clinical cases that required clinical pharmacy input (reported in five hospitals). Following the national AMS guidelines, clinical pharmacists were required to develop specific antibiotic use guidelines and training and to monitor antibiotic use and compliance. These roles and responsibilities were considered challenging by the participating pharmacists:

*“Clinical pharmacy recently received more support and made more contacts with doctors … although it differs per clinical pharmacist. To reach the expected level for clinical pharmacists we need to increase their capacity because most of them are young with limited experience in order for them to communicate with our ID doctors who have years of clinical experience.” (IDI, quality control/emergency doctor, male, 35 years old)*

Overall, there is a gap in clinical interactions between microbiology, pharmacy and clinical departments in all study hospitals with the exception of the hospital with the most active AMS programme:

*“We need to improve the collaboration between microbiology, pharmacy and clinical departments for more effective antimicrobial management. Currently, the difficulties are more from microbiology, they lack resources to participate in specialised issues, lack of connection with doctors and clinical pharmacists, so this area is still left behind.” (FGD, senior staff)*

#### 3.2.4 Education and training

There is no formal training for AMS either at undergraduate or postgraduate level for medical professionals and pharmacists in Viet Nam. The participants expressed the need for training on clinical pharmacy (for pharmacists), updates on new drugs and new pathogens, local AMR patterns, research evidence on the quality of generic drugs, clinical microbiology (for microbiologists), infection diagnosis, antimicrobial drug interactions and incompatibility/effective combination therapies for doctors, and infection control.

#### 3.2.5 Other specific actions aiming at responsible antimicrobial use

Two of the seven hospitals developed hospital-specific antibiotic treatment guidelines based on local evidence. At one hospital with successful implementation of treatment guidelines, strong leadership commitment and support for the AMS team was considered to be the driving factor.

Despite the fact that not all hospitals established their own AMS teams, all study hospitals applied the guidance on antibiotic pre-authorisation policy as recommended in the national AMS guideline, partly also for insurance reimbursement requirements. Prospective audit and feedback to prescribers were performed in two hospitals in Group 2, and retrospective review of antimicrobial prescriptions was done on an ad hoc basis in all hospitals.

#### 3.2.6 Monitoring and surveillance/reporting and feedback

In all hospitals, the pharmacy department only monitored data on the amount of antibiotics supplied to clinical wards, not the actual antibiotic administration. Appropriateness of antibiotic use was monitored through retrospective review of medical charts on an ad hoc basis in all hospitals and through prospective audit and feedback activity in two hospitals in Group 2; results were reported to hospital leaders and heads of clinical wards and occasionally shared at national/regional meetings. Common issues discussed by the participants in monitoring antimicrobial use included lack of staff to review medical charts and no software to support this activity.

### 3.3 Survey on perceptions and attitudes among staff

**Fig. 3:**
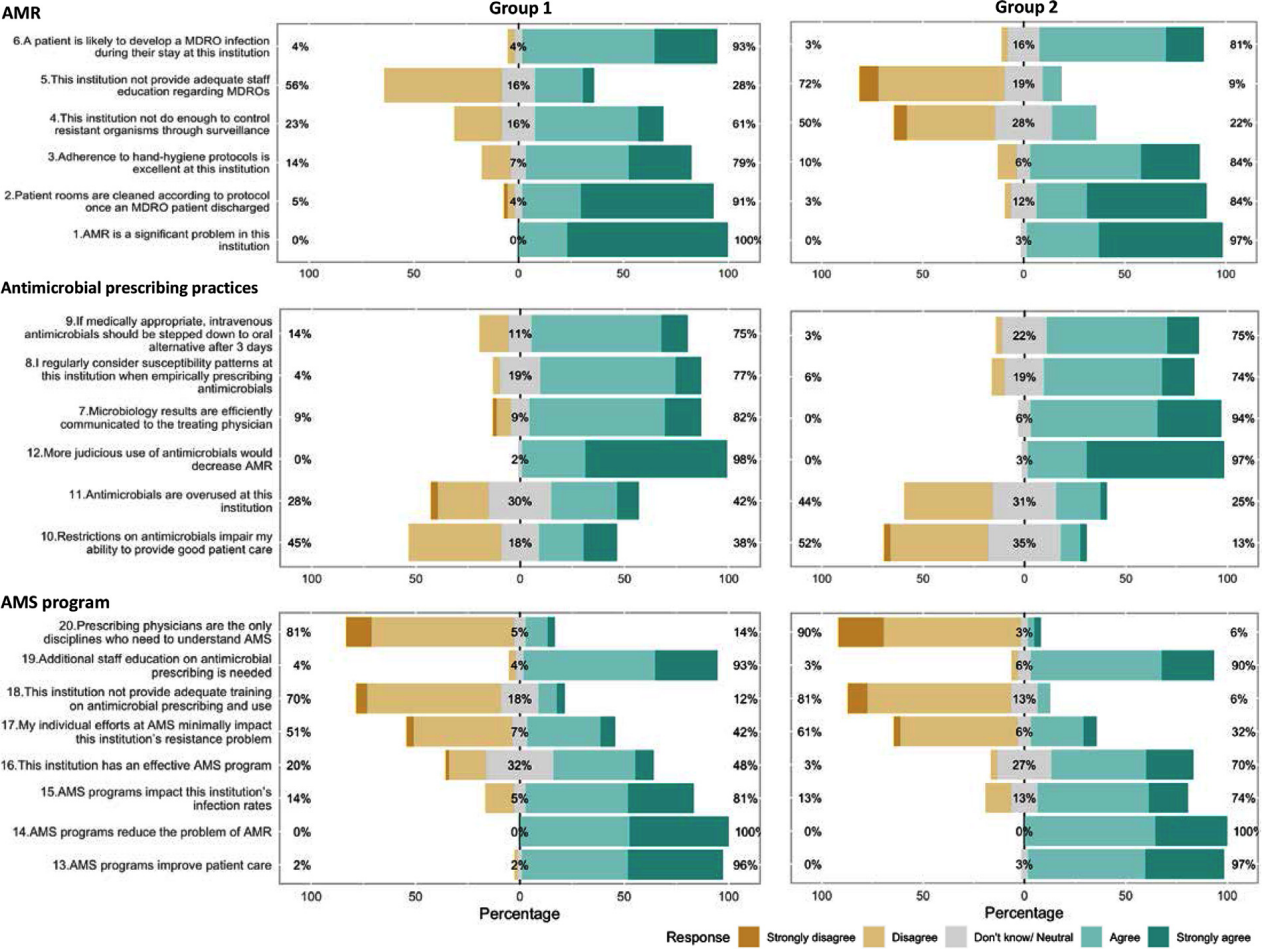
Staff perceptions about antimicrobial resistance (AMR), antimicrobial prescribing and antimicrobial stewardship (AMS) at their hospital. MDRO, multidrug-resistant organisms.

The survey results overall showed similarity among staff perceptions regarding AMR, prescribing practices and AMS programmes between hospitals and between groups 1 and 2 (Fig. 3). In both groups, staff largely agreed that AMR was an important problem at their institution, that adherence to IPC procedures was excellent, that AMS was positive for patient care and controlling AMR, and that they adhered to the intravenous–oral switch.

Although both groups stated that they considered local susceptibility patterns when empirically prescribing antimicrobials (44/57 vs. 23/33; *P* = 0.59), more participants in Group 1 agreed that their hospitals did not do enough to control AMR through surveillance (35/57 vs. 7/33; *P* = 0.00053). Similarly, Group 1 participants were more likely to agree that their hospital did not provide adequate staff education and training on multidrug-resistant organisms, but this difference was not statistically significant (16/57 vs. 3/33; *P* = 0.063). Participants in Group 1 were more likely to consider that restrictions on antimicrobials impaired their ability to provide good patient care (21/57 vs. 4/33; *P* = 0.023). More participants in Group 1 also disagreed with the statement that their hospital had an effective AMS programme, although this difference did not reach statistical significance (11/57 vs. 1/33; *P* = 0.0062).

When asked about the perceived levels of resistance in their institution for common bacterial pathogens, the responses varied widely among the surveyed staff in all study hospitals (Supplementary Fig. S1). Among the nine bug–drug combinations, the mean amount of divergence in the perceived levels in comparison with the measured proportions from surveillance was highest for *Acinetobacter baumannii–*carbapenem [31.39%, 95% confidence interval (CI) 25.08–37.71%], followed by *Escherichia coli–*third-generation cephalosporin (29.77%, 95% CI 24.80–34.74%) and methicillin-resistant *Staphylococcus aureus* (29.26%, 95% CI 24.43–34.08%) and lowest for vancomycin-resistant enterococci (17.03%, 95% CI 11.94–22.12%) (Table 3). Results from the multivariable mixed-effects model showed that staff working in surgery were more likely to give an incorrect proportion of resistance (compared with the measured proportion from surveillance) with the highest amount of divergence (36.38% 95% CI 29.53–43.23%) among the departments. Working in a hospital with an established AMS programme (Group 2) did not significantly influence the amount of divergence in the perceived compared with the measured proportions of resistant organisms.

**Table 3:** Factors associated with the amount of divergence in staff's perceived proportion of resistance in comparison with reported proportion of resistance from the surveillance data; results from a multivariable mixed-effects model for perceived resistant proportions of all bacteria–antibiotic combinations

Factor	Mean (95% CI) group value (%) a	Model coefficient b	*P-*value
Study group			
Group 1	26.21 (24.22–28.20)	Ref.	
Group 2	22.60 (19.79–25.41)	–1.41	0.88
Age of participants	–	–0.18 c	0.72
Bacteria–antibiotic combination			
*Escherichia coli–*third-generation cephalosporin	29.77 (24.80–34.74)	Ref.	
*Klebsiella pneumoniae*–carbapenem	17.24 (13.72–20.75)	–13.17	0.00052
*Klebsiella pneumoniae*–third-generation cephalosporin	23.01 (19.13–26.89)	–6.91	0.070
*Pseudomonas aeruginosa–*carbapenem	26.91 (22.67–31.16)	–3.91	0.30
*Pseudomonas aeruginosa–*ceftazidime	22.40 (17.18–27.62)	–6.77	0.080
*Pseudomonas aeruginosa–*ciprofloxacin	26.63 (22.32–30.94)	–1.22	0.75
*Acinetobacter baumannii–*carbapenem	31.39 (25.08–37.71)	4.81	0.20
Methicillin-resistant *Staphylococcus aureus*	29.26 (24.43–34.08)	0.79	0.84
Vancomycin-resistant enterococci	17.03 (11.94–22.12)	–16.46	<0.0001
Years of work at the hospital			
>20 years	19.05 (14.54–23.56)	Ref.	
16–20 years	27.23 (23.43–31.03)	8.62	0.37
11–15 years	25.52 (21.61–29.43)	14.95	0.19
6–10 years	24.54 (21.84–27.24)	10.01	0.36
1–5 years	22.39 (18.71–26.06)	8.52	0.54
<1 year	35.78 (25.77–45.79)	18.06	0.26
Department			
ICU	24.85 (21.91–27.79)	Ref.	
Internal medicine	27.94 (24.94–30.94)	5.20	0.21
Surgery	36.38 (29.53–43.23)	17.88	0.09
Microbiology	17.87 (14.34–21.40)	–6.55	0.23
Pharmacy	18.51 (13.63–23.39)	–9.54	0.21
Others	22.59 (18.11–27.07)	0.06	0.99

CI, confidence interval; ICU, intensive care unit.

NOTE: Linear mixed model was fit using the *lmer* function in *lmerTest* package in R program v.4.0.0.

a Mean group value represents the amount of divergence in staff's perceived proportion of resistance in comparison with the reported proportion of resistance from the surveillance data.

b Model coefficient represents the difference in the amount of divergence between the group of interest in comparison with the reference group under each factor.

c For every year increase in age.

## 4 Discussion

Here we describe the factors associated with implementation of AMS programmes in 7 of 16 hospitals in the VINARES network in Viet Nam [14]. Our findings show a complex matrix of factors and actors that can influence AMS activities and ultimately doctors’ prescribing practices, from the national to the local level. Our survey also shows that AMS programmes were perceived positively by hospital staff in providing an impact on patient care and controlling AMR. The uptake and impact of AMS programmes varied greatly among the hospitals in this study, as was also reported in a national quantitative survey [17]. This reflects the fact that implementation was highly contextual and reliant on local leadership commitment, local resources and capacity, and the professional characteristics and interactions in each institution. In addition, our study confirms that the composition of the AMS team and collaboration between different departments determine the success of AMS programme activities [25].

Although variations exist, in many AMS programmes the integral roles of clinical microbiologists and clinical pharmacists are emphasised [27]. However, communication on antimicrobial prescribing still remains mostly between clinical doctors in our study, while interactions with microbiology or pharmacy are limited to traditionally defined roles (providing information on test results and drugs). Both clinical microbiologists and pharmacists lack clinical training, experience and self-confidence to debate with clinical doctors about antimicrobial prescribing. This is a common challenge for AMS implementation in LMIC hospital settings [28]. There was also a lack of understanding by doctors on what clinical microbiology can provide to support them in antimicrobial treatment. Importantly, doctors need to have more understanding of local cumulative susceptibility data and be able to use this evidence to guide empirical treatment.

The role of clinical pharmacists has received more policy attention in recent years as evidenced by the 2016 Vietnamese Pharmacy Law. This law requires clinical pharmacists to review prescriptions for medications in their hospital, examine the patient medical records, and report to the director board [29]. The core AMS team recommended in the MoH updated guideline includes only two roles: clinical doctors and clinical pharmacist [16]. Fulfilling the AMS responsibilities with the current capacity of clinical pharmacists is a challenge for the hospitals in this study. Future policy directions should clearly aim to improve the staffing capacity for AMS in hospital, from providing training options in the formal education programmes to establishing sufficient staffing standards for hospitals in the country.

The AMS staffing recommendation in high-income countries is around two to six full-time equivalents per 1000 acute-care beds [30]. The regional consensus statement on AMS in Asia [31] recommends having an infectious diseases specialist leading the programme with day-to-day support from clinical pharmacists in a multidisciplinary team, which also includes a clinical microbiologist, clinicians with expertise in IPC and epidemiology, and information technology experts. The amount of reported hours dedicated to AMS programmes per week in a recent international survey was on average 13, 8 and 6 h for pharmacist, infectious diseases doctor and clinical microbiologist, respectively, for hospitals in Asia (*n* = 25) compared with 32, 15 and 5 h in North America (*n* = 49) and 18, 8 and 11 h in Europe (*n* = 190) [18]. The recently issued recommendation in Viet Nam for the core AMS team is to have at least three staff in large hospitals and one staff in small hospitals (clinical doctors and/or clinical pharmacists) (not specifying whether or not these staff should be full-time) [16]. More specific policy mechanisms are still required to enable hospitals in following such recommendations to effectively implement AMS activities.

The main strength of this mixed-methods study is that we included a wide range of hospital staff from different clinical and non-clinical departments and included hospitals with active and less-active AMS programmes to participate in the study to obtain a comprehensive picture of AMS implementation in Viet Nam. We also included multiple data collection methods and recruited both experienced and junior staff to ensure the richness and cross-validation of data. Nonetheless, there are several limitations that need to be discussed. First, we included hospitals based on their willingness to participate in this study, therefore the participating hospitals were likely to have higher levels of interest in AMS implementation than those refusing participation. In addition, we only contacted hospitals in the VINARES network, which consists of national and provincial level hospitals involved in AMR surveillance that receive support from government and international funders and organisations [14,32,33]. Therefore, the findings may not be representative of all provincial and national level hospitals. Furthermore, our sample did not include district level hospitals (684/1150 public hospitals in Viet Nam [34]). Further research will also be required to quantitatively evaluate the impact and cost effectiveness of AMS programmes in order to provide more concrete evidence to support AMS planning and implementation for hospitals in Viet Nam and other LMICs.

## 5 Conclusions

AMS programmes have been implemented at the studied hospitals in response to MoH guidelines, although at varying levels of leadership commitment and staff engagement. A higher level of leadership support and commitment to AMS has led to a higher level of engagement in AMS activities from the AMS teams and effective collaboration between departments involved. There is a good opportunity for hospitals in implementing AMS programmes as the impact of such AMS programmes on patient care and AMR are perceived positively by staff. Building AMS staffing capacity tailored to local conditions, creating recommendations on how AMS staff can be effectively funded, and developing standards for AMS education and training are urgently needed to help improve programme implementation in hospitals in Viet Nam and similar LMICs.

## Appendix Supplementary materials

Image, application 1
