# Supplementary material for: Improving antimicrobial use through antimicrobial stewardship in a lower-middle income setting: a mixed-methods study in a network of acute-care hospitals in Viet Nam
Source: J Glob Antimicrob Resist. 2021 Dec;27:212–21. doi: 10.1016/j.jgar.2021.09.006 (PMC8692234; doi:10.1016/j.jgar.2021.09.006)
Supplement: Supplementary file 1 [file mmc1.docx]

**Supplementary File for manuscript**

**Article Title**

**Improving antimicrobial use through antimicrobial stewardship in a lower-middle income setting: a mixed methods study in a network of acute-care hospitals in Viet Nam**

**Supplementary Methods:**

**A. Question Guide for In-depth Interview**

*(For use at hospitals where antimicrobial stewardship team has been established)*

1. How often do you prescribe antimicrobials for your patients? What sorts of patients will you decide to use antimicrobials? What is the process that you follow in prescribing antimicrobials throughout the patient care and treatment? (*prompts: empirical therapy, performing diagnostics, review continuing antimicrobial need, changes in antimicrobial decisions, documenting antimicrobial decisions*)
2. How is patient information communicated between you and following staff at your hospital in relation to diagnostics and antimicrobial treatment? Have you had any difficulties in this communication? If yes, what are the difficulties? (*prompts for details, i.e. completeness, accuracy, clinical relevance*)

- Other staff in the ward round
- Lab staff
- Pharmacy
- Senior clinician/ manager

Based on the checklist on ASP (Appendix A), identify with the participant the components that have been implemented at their hospital.

1. Are the ASP strategies implemented at your hospital (*ask for each of the implemented strategies*) effective in the following aspects:

- improving patient clinical care and outcomes
- improving antimicrobial prescribing practice
- reducing antimicrobial resistance situation

Please give examples on how these have been effective or not effective.

Which ASP strategies have been most effective and why? *(Prompts: How did you prescribe before the ASP? What are the changes in your practice after implementation of ASP in this hospital? What activities have influenced your prescribing behavior the most? What activities have the greatest impact on patient clinical care and outcomes? What activities have the greatest impact on resistance rates at your hospital?)*

1. What are the factors that contribute to the success in the implementation of ASP strategies (*ask for each implemented strategy*)? What are the barriers or difficulties occurring during the implementation? Please give specific examples from your experience.
2. Some doctors say that the ASP takes away some of their prescribing autonomy and interferes with their clinical decision making. Do you think that the ASP has also affected you in the same way? Please describe your experience.
3. What are the specific trainings that have been organized at this hospital as part of the antimicrobial stewardship program or aiming to improve antimicrobial prescribing? Please give details on frequency and topics of each training.

Apart from these on-site trainings, have you attended any other training related to antimicrobial stewardship or antimicrobial prescribing? What areas do you think more trainings should be provided on to the clinicians/ lab staff/ pharmacists/ other hospital staff?

**B. Guide for Focus Group Discussion**

| Type of question | Content |
| --- | --- |
| OPENING  (5 minutes) | - Tell us who you are, what your background by training is, how long you have been working for this hospital, what your current work position is, and how long you have been in this position for at this hospital. |
| INTRODUCTION  (10 minutes) | - What is the first thing that comes to mind when you hear the word “antimicrobial resistance”? Why is this important? What are the reasons for antimicrobial resistance at your hospital? |
| TRANSITION  (10 minutes) | - Have you heard of the antimicrobial stewardship program? Please tell us what you understand about this program? Do you know about the ASP Guidelines issued by the MoH?   *Summarise the main aspects of MoH’s ASP Guidelines.* |
| KEY  (60 minutes) | - Has ASP been discussed at this hospital? If yes, at which levels? Has the hospital made any plan for adopting ASP? If yes, what is the plan? - Imagine an ASP will be implemented at your hospital:   + Who should lead and be responsible for monitoring and managing the program? Who should be part in the ASP team?  + What strategies should be part of the ASP at this hospital? (*Show a list of recommended strategies*). Please give reasons why or why not.  + How should the program be monitored? Which indicators?   - What are the supportive factors for the adoption of ASP at your hospital? What are the negative factors against the adoption of ASP at your hospital? (*prompts:* leadership/ policy/ financial/ human/ physical resources/communications between departments and wards/staff knowledge/ awareness/ attitudes) |
| ENDING  (10 minutes) | *Moderator summarizes the main content of the focus group.*   - Is there anything else you would like to add or mention before we wrap- up? |

**C. Antimicrobial stewardship perception survey**

(Based on Antimicrobial Stewardship Toolkit 2011 by United Hospital Fund, Greater New York Hospital Association: <https://uhfnyc.org/publications/publication/antimicrobial-stewardship-toolkit/>)

Please indicate your agreement or disagreement with the following statements about your institution.

| **ANTIMICROBIAL RESISTANCE: SCOPE OF THE PROBLEM AND KEY CONTRIBUTORS** | | | | | | | |
| --- | --- | --- | --- | --- | --- | --- | --- |
|  | Strongly Disagree | Disagree | Neither | Agree | Strongly Agree | | Don’t know |
| 1. Antimicrobial resistance is a significant problem in this institution. |  |  |  |  |  | |  |
| 2. Patient rooms are cleaned according to hospital cleaning protocol once a multidrug resistant organism (MDRO) patient has been discharged. |  |  |  |  |  | |  |
| 3. Adherence to hand-hygiene protocols is excellent at this institution. |  |  |  |  |  | |  |
| 4. This institution does NOT do enough to control the develop­ment of resistant organisms through surveillance. |  |  |  |  |  | |  |
| 5. This institution does NOT provide adequate staff education regarding MDROs. |  |  |  |  |  | |  |
| 6. A patient is likely to develop a MDRO infection during their stay at this institution. |  |  |  |  |  | |  |
| **ANTIMICROBIAL PRESCRIBING PRACTICES** | | | | | | | |
| 7. Microbiology lab results are efficiently communicated to the treating physician. |  |  |  |  |  |  | |
| 8. I regularly refer to/consider the antimicrobial susceptibility pat­terns at this institution (e.g., the institutional antibiogram) when empirically prescribing antimicrobials. |  |  |  |  |  |  | |
| 9. If medically appropriate, intravenous antimicrobials should be stepped down to an oral alternative after three days. |  |  |  |  |  |  | |
| 10. Restrictions on antimicrobials impair my ability to provide good patient care. |  |  |  |  |  |  | |
| 11. Antimicrobials are overused at this institution. |  |  |  |  |  |  | |
| 12. More judicious use of antimicrobials would decrease antimicrobial resistance. |  |  |  |  |  |  | |
| **ANTIMICROBIAL STEWARDSHIP PROGRAMS**  (A formal program that monitors and manages the appropriate use of antimicrobials.) | | | | | | | |
| 13. Antimicrobial stewardship programs improve patient care. |  |  |  |  |  |  | |
| 14. Antimicrobial stewardship programs reduce the problem of antimicrobial resistance. |  |  |  |  |  |  | |
| 15. Antimicrobial stewardship programs impact this institution’s infection rates. |  |  |  |  |  |  | |
| 16. This institution has an effective antimicrobial stewardship program. |  |  |  |  |  |  | |
| 17. My individual efforts at antimicrobial stewardship minimally impact this institution’s resistance problem. |  |  |  |  |  |  | |
| 18. This institution does NOT provide adequate training on anti­microbial prescribing and use. |  |  |  |  |  |  | |
| 19. Additional staff education on antimicrobial prescribing is needed. |  |  |  |  |  |  | |
| 20. Prescribing physicians are the only disciplines who need to understand antimicrobial stewardship. |  |  |  |  |  |  | |

**PREVALENCE OF ANTIMICROBIAL RESISTANCE**

Please indicate the proportion of isolates of each organism listed below that is resistant to the antimicrobial indicated at your institution. You are not expected to know the exact figure, we are interested in your best guess.

| *Staphylococcus aureus* resistant to methicillin or oxacillin (i.e., MRSA) | % |
| --- | --- |
| *Enterococcus faecium* resistant to vancomycin (i.e., VRE) | % |
| *Pseudomonas aeruginosa* resistant to ciprofloxacin | % |
| *Pseudomonas aeruginosa* resistant to ceftazidime | % |
| *Pseudomonas aeruginosa* resistant to carbapenems | % |
| *Klebsiella pneumoniae* resistant to 3GC | % |
| *Klebsiella pneumoniae* resistant to carbapenems | % |
| *E. coli* resistant to 3GC | % |
| *Acinetobacter baumannii* resistant to carbapenems | % |

**BACKGROUND INFORMATION**

1. What is your primary work area or unit in this institution? (Please check ONE )

| ☐ Many different units/ No specific unit | ☐ Medicine (non-surgical) | ☐ Intensive care unit (any type) | ☐ Radiology |
| --- | --- | --- | --- |
| ☐ Surgery | ☐ Psychiatry/mental health | ☐ Obstetrics | ☐ Rehabilitation |
| ☐ Anesthesiology | ☐ Pediatrics | ☐ Pharmacy | ☐ Laboratory |
| ☐Emergency department | | ☐ Other (please specify): | |

2. How long have you worked in this institution?

| ☐ Less than 1 year | ☐ 6 to 10 years | ☐ 16 to 20 years |
| --- | --- | --- |
| ☐ 1 to 5 years | ☐ 11 to 15 years | ☐ 21 years or more |

3. What is your staff position in this institution?

| ☐ Attending/Staff physician | ☐ Specialist | ☐ Physician assistant | ☐ Infection control practitioner |
| --- | --- | --- | --- |
| ☐ Resident physician/Intern | ☐ Pharmacist | ☐ Nurse practitioner | ☐ Other (please specify): |

4. How long have you worked in your current specialty or profession?

| ☐ Less than 1 year | ☐ 6 to 10 years | ☐ 16 to 20 years |
| --- | --- | --- |
| ☐ 1 to 5 years | ☐ 11 to 15 years | ☐ 21 years or more |

**Supplementary Tables:**

**Supplementary Table 1.** Additional representative quotes from in-depth interviews and focus group discussions at the seven study hospitals

| **Theme** | **Representative quotes** | **Source of quote** |
| --- | --- | --- |
| Leadership | If it is not compulsory, nobody would call us. But when the AMS committee or DTC make it as a policy requesting consultations with clinical pharmacists and microbiologists, they need to invite us and seek our comments written in the medical records. In our procedure for reviewing and approving antimicrobial prescriptions, it is compulsory that pharmacy need to review before the head of planning department and director-board review. This increases our status quo. | IDI-H3-AMS coordinator-Pharmacy-female, 34 years old |
|  | I think in order to establish such a (AMS) committee, we firstly would need to have the support from the hospital directory board and the directory board should issue the decision about staff. Without the support and approval from the hospital directory board, it would be difficult to implement such a program. | FGD-H1-Senior staff |
|  | The directors do not pay much attention to this because of the small amount of expenditure for antibiotics, although there are many problems with antibiotic treatment practices here but the amount we use is so mall compared to other hospitals. | FGD-H7-Senior staff |
| Collaboration | The planning department needs to review any conflicts between clinical pharmacists and doctors. Sometimes he agreed with the clinical pharmacist, some other times he agreed with the treating doctor. Comments from clinical pharmacists are thus to support the final decision making process. | IDI-H3-AMS coordinator-Pharmacy- female, 34 years old |
|  | Meetings focusing on developing guidelines on specific clinical areas the head of corresponding departments had to attend because they need to know how the guidelines would be applied in their departments… This sometimes need meetings every 2 weeks and required up to 8 meetings in total to make a decision. | IDI-H3-AMS coordinator-Pharmacy- female, 34 years old |
| De-escalation | The aim is to make treatment duration as short as possible. For example when microbiology results come back on day 3 or 4 of treatment, there is no reason for us to de-escalate to a narrower antimicrobial and add another 3-4 days of treatment… In this case, we only need to continue the existing antimicrobial for another day and then we stop. Or when patient is still severe with fever we would not de-escalate despite microbiology results should susceptibility to a narrower antimicrobial… And generally there are not reasons to de-escalate if we have good clinical response and in absence of AST results. | IDI-H3-ICU- female, 29 years old |
|  | Health insurance limit, for example will pay up to 7 million dongs for the patient. Some narrow-spectrum drugs are expensive… so we need to consider about this given the insurance limit. | IDI-H6-Tropical Medicine-male, 43 years old |
|  | If we use oral drugs, health insurance will ask that the patient can be treated as an outpatient… Outpatient treatment expenses need to be covered by the patient out-of-pocket. | IDU-H6-Planning Department- female, 42 years old |
|  | De-escalation is rare in our department. One difficulty is doctors’ psychology. For example, with current antimicrobials, the patient was progressing well, but now if we switched to oral drugs or applied de-escalation, what would happen to the patient? What if the patient could not absorb the drugs? What if after 1 or 2 days of de-escalation, fever came back to the children?... Working in clinical area is quite difficult. | FGD-H7-Junior staff |
| Drugs | | |
| Brand-name versus generic | If the case is too severe and we cannot rule out CNS infection we tend to use Meronem more then Imipenem if we can assess CSF. About Ceftriaxone if it is really a CNS infection I still like to use brand-name drug. I use generic drugs in common, non-life threatening, non-sepsis infections…  …We have many restrictions on drugs, for severe patients our doctors do not have the habit to trust generic drugs, no strong evidence to support the effectiveness of generic drugs. Our doctors only have information from available resources mostly for brand-name drugs, we don’t know much about drug interactions (for generic drugs)… | IDI-H3-ICU- female, 29 years old |
|  | Drugs are available in the drug list but sometimes the bidding law only permit us to have certain amount of drugs. We have to use alternatives if a drug is out of stock. In Vietnam, brand-name and generic drugs are mixed. We don’t know about the actual quality. We can’t verify or trust drug quality. | FGD-H5-junior staff |
| IV to PO | I think when patients are hospitalized they prefer IV. If we say they can take oral drugs, they seem not to like and say they can go home and take oral drugs. | IDI-H4-Respiratory Internalist-male, 39 years old |
| Drug quality | We use whatever available in the formularies, using but worrying at the same time, indeed... | IDI-H4-Respiratory Internalist-male, 39 years old |
|  | Four-five years ago, it was good because we used brand-name drugs, even Vietnamese drugs were also better. Now so many drugs, and we ourselves do not control the quality, so it is difficult. We sometimes talked with each other during ward rounds about this drug or that drug did not work so well even when we doubled the dose… | IDI-H6-Surgical ICU-female, 37 years old |
| Restricted antimicrobials | The approval system for restricted antimicrobials is useful, but sometimes delays antimicrobial use in severe cases for up to 30 minutes or 1 hour. Although I think this approval is appropriate to avoid overuse. | IDI-H4-ICU doctor, male, 32 years old |
| Prospective audit with feedback to prescribers | It is very rare to have good collaboration between pharmacists and doctors from the beginning. We had to allow some time when we go with them, coupled with the support from the leaders emphasizing that clinical pharmacists are not checking on the wards to point out errors but to support the wards. I had to be thoughtful, initially just giving recommendations but not documenting anything so they grow trust in us and then share with us… It is an art. | IDI-H3-AMS coordinator-Pharmacy- female, 34 years old |
| Microbiology | AST results are often available after 3 days. It’s better to have AST but it is late. In non-severe cases I will continue to treat (the empiric therapy). In severe cases I will consider the benefits and consequences and make decision on a combination therapy, I don’t need AST results. | IDI-H4-Gastroenteritis Internalist- female, 45 years old |
|  | We do not have a lot of multi-drug resistance problem in our department so we don’t have as much demand for microbiology as in ICU. | IDI-H4-Gastroenteritis Internalist- female, 45 years old |
|  | Cascading report was implemented based on the principle that treating doctors are still the one who makes the final decision on treatment because patient treatment does not only depend on microbiology results… After consulting with microbiology and pharmacy and even with the planning department, (the doctor) select the best treatment option most appropriate for the patient. | IDI-H3-Internal Medicine- female, 47 years old |
|  | If a patient has infections, we will treat based on antimicrobial spectrum and our experience. It usually works, but if the patient isn’t better after 3 days of treatment, we’ll do culture test. | FGD-H7-Senior staff |
|  | Clinical doctors have to connect with microbiology, and microbiology has to increase clinical exposure. And this interactive 2-way communication is currently very weak. | IDI-H5-Quality Control/Emergency-male, 35 years old |
|  | Provide data to convince doctors. For example, after being given susceptibility evidence for Piperacillin, doctors started to have more confidence and using this drug. | IDI-H3-Internal Medicine- female, 47 years old |
| Guidelines | I usually follow international treatment guidelines, and in Vietnam I follow guidelines of Bach Mai and Cho Ray | IDI-H4-Gastroenteritis Internalist-female, 45 years old |
|  | MoH guideline does not cover all specific clinical cases so we rarely follow. Mainly find and read international guidelines, communicate with colleagues, and based on our own individual experience to treat. | Communication with doctors during Observation H4 02Aug18-Respiratory Neurology and Endocrinology |
|  | Even though some related activities have been implemented under the Drug and Therapy Committee, they have been separated under the AMS Committee since 2016 when the MoH Decision 772 with the AMS guideline… This guideline provides clear guidance on members and planning activities. | IDI-H3-AMS coordinator- female, 34 years old |
| Documentation of treatment reasons | Our doctors are usually not good at documenting reasons for de-escalation… He can state something like culture results show bacteria A and AST show susceptibility to antimicrobial B and C, which is very simple. But doctors do not document, even though when they practiced correctly but were lazy to write. | IDI-H5-Quality Control and ICU doctor-male, 35 years old |
|  | We usually write in the medical record that culture results were returned with the AST results and that we adjust antibiotics based on the AST results… We treat with the duration based on what we learnt, we don’t write the anticipated stop date. | IDI-H4-ICU doctor-male, 32 years old |
| Monitoring and Reporting | We have done DDD analysis for 6 months. In fact we have put lots of efforts. Not much interest and too time-consuming. Sometimes doctors do not know what 30 means and how to conclude, not clear about the meaning of DDD. | FGD-H7-Junior staff |
|  | No evaluation here because doctors are hesitant about appropriateness of prescriptions being monitored and there are insufficient clinical pharmacists available to monitor. | FGD-H2-senior staff |

**Supplementary Table 2.** Number of responses on staff perceptions about antimicrobial resistance (AMR), antimicrobial prescribing, and antimicrobial stewardship (AMS) at their hospital

|  | Group 1_(n=4, 57 participants) | | | Group 2 (n=3, 33 participants) | | |
| --- | --- | --- | --- | --- | --- | --- |
|  | D | A | O | D | A | O |
| 1. Antimicrobial resistance is a significant problem in this institution. | 0 | 56 | 1 | 0 | 30 | 3 |
| 2. Patient rooms are cleaned according to hospital cleaning protocol once a multidrug resistant organism (MDRO) patient has been discharged. | 3 | 52 | 2 | 1 | 27 | 5 |
| 3. Adherence to hand-hygiene protocols is excellent at this institution. | 8 | 45 | 4 | 3 | 26 | 4 |
| 4. This institution does not do enough to control the develop­ment of resistant organisms through surveillance. | 13 | 35 | 9 | 16 | 7 | 10 |
| 5. This institution does not provide adequate staff education regarding MDROs. | 32 | 16 | 9 | 23 | 3 | 7 |
| 6. A patient is likely to develop a MDRO infection during their stay at this institution. | 2 | 53 | 2 | 1 | 26 | 6 |
| 7. Microbiology lab results are efficiently communicated to the treating physician. | 5 | 47 | 5 | 0 | 30 | 3 |
| 8. I regularly refer to/consider the antimicrobial susceptibility pat­terns at this institution (e.g., the institutional antibiogram) when empirically prescribing antimicrobials. | 2 | 44 | 11 | 2 | 23 | 8 |
| 9. If medically appropriate, intravenous antimicrobials should be stepped down to an oral alternative after three days. | 8 | 42 | 7 | 1 | 24 | 8 |
| 10. Restrictions on antimicrobials impair my ability to provide good patient care. | 25 | 21 | 11 | 16 | 4 | 13 |
| 11. Antimicrobials are overused at this institution. | 16 | 24 | 17 | 14 | 8 | 11 |
| 12. More judicious use of antimicrobials would decrease antimicrobial resistance. | 0 | 55 | 2 | 0 | 30 | 3 |
| 13. AMS programs improve patient care. | 1 | 55 | 1 | 0 | 30 | 3 |
| 14. AMS programs reduce the problem of AMR. | 0 | 57 | 0 | 0 | 31 | 2 |
| 15. AMS programs impact this institution’s infection rates. | 8 | 46 | 3 | 4 | 23 | 6 |
| 16. This institution has an effective AMS program. | 11 | 27 | 19 | 1 | 21 | 11 |
| 17. My individual efforts at AMS minimally impact this institution’s resistance problem. | 29 | 24 | 4 | 19 | 10 | 4 |
| 18. This institution does not provide adequate training on anti­microbial prescribing and use. | 39 | 7 | 11 | 25 | 2 | 6 |
| 19. Additional staff education on antimicrobial prescribing is needed. | 2 | 53 | 2 | 1 | 28 | 4 |
| 20. Prescribing physicians are the only disciplines who need to understand AMS. | 46 | 8 | 3 | 28 | 2 | 3 |

D: Disagree; A: Agree; O: Others (no comment/don’t know/missing)

**Supplementary Figure 1. Perceived levels of resistance among the surveyed staff at their local hospital;** Each graph is for one hospital in Group 1 or Group 2; Each dot represents the response of each participant about the resistant proportion (%) for each listed pathogen-antimicrobial combination. Each asterisk represents the estimated hospital-wide resistant proportion for each combination at each hospital from the surveillance data for all types of specimens in 2016.
